# Financial strain of COVID-19 and its impact on willingness-to-pay for equine care

**DOI:** 10.1093/jas/skad091

**Published:** 2023-03-26

**Authors:** Jada M Thompson, Michelle L Kibler, Jennie L Z Ivey

**Affiliations:** Department of Agricultural Economics and Agribusiness, University of Arkansas, Fayetteville, AR, USA; Department of Agriculture, Illinois State University, Normal, IL, USA; Department of Animal Science, University of Tennessee, Knoxville, TN, USA

**Keywords:** board, double bounded dichotomous choice, equine, lease

## Abstract

The novel COVID-19 virus caused a global pandemic disrupting lives, industries, and economies. The result was an impact on prices due to challenges with production and supply chain distribution. This study investigates the financial strain COVID-19 had on equine owners and leasers, what the market for equine care would bear if costs for care increased, and what factors contribute to their willingness to pay (**WTP**) for increasing cost of equine care. An online survey was distributed for 4 weeks to adult U.S. residents. Respondents reported their involvement in the equine industry, financial response to COVID-19, and responses to a double-bound dichotomous choice question on their WTP for care given a randomized increase (1%–20%) in their current cost (*n* = 506). Data were analyzed using interval regression models where *a* = 0.05 (Stata15). Respondents were separated into three groups: owner keeping their horse at their residence, owner boarding their equids, and leaser. Boarders reported mean monthly payments of 23.33 ± 90.37 USD (*n* = 15) for free board, 236.47 ± 151.92 USD, (*n* = 75) for partial board, and 514.75 ± 291.71 USD (*n* = 181) for full board. Results show all owners, leaser, and boarders have different WTP values which range from 18.5% to 26.2% increase in current care costs which extends beyond the presented range due to many respondents responding “yes” to both WTP questions (71% of owners, 6% of boarders, 65% of leasers). Equine owners, with on farm equids from the southern US were WTP 11% less than from other regions (*P* = 0.015). The current boarding fees from owners that board their equids lowered their WTP by 0.01% (*P* = 0.029) for each additional dollar paid, whereas current care costs were not a significant factor for other owners (*P* = 0.370) or leasers (*P* = 0.395). Those that had a full lease for their equids, housed on farm or at a facility, were WTP 15% higher (*P* = 0.036) than those that had a partial or no lease. In comparison, boarding status (full, partial, or no boarding) did not significantly (*P* = 0.51) impact boarder’s WTP. Age of respondent and annual household income heterogeneously affected WTP across all groups. These results indicate the market for equine care can bear the increases in cost associated with financial distress related to COVID-19, and may aid equine owners, caregivers, and associated individuals in making informed decisions regarding essential care. Results from this study should be taken in context of the global pandemic and the restrictions in place, or lack thereof, at the time the survey was administered.

## Teaser

Essential and nonessential equine care is important to owner and leasers of equids. They are willing to increase the amount paid for care even in the middle of financial distress.

## Introduction

The Centers for Disease Control (**CDC**) and the World Health Organization (**WHO**) both declared the emergent SARS-CoV-2 (**COVID-19**) a global pandemic in March 2020 which was quickly followed by nationwide lockdowns and policies intended to limit the spread of the virus around the world. What followed affected the global economy, brought record unemployment levels to 44 out of the 50 U.S. states (U.S. Bureau of Labor Statistics, 2021), disrupted supply chains (Maples et al., 2021; Weersink et al., 2021), affected food security (Ahn and Norwood, 2021), and led to volatile prices for goods and services (Ellison et al., 2021; Ruan et al., 2021). These impacts were felt in the livestock industry through backups at meat processing facilities and changing consumer demand for meat consumed at home vs. away from home. Conversely, the demand for companion animals increased as a result of the social quarantine policies established to slow the spread of the pandemic leaving many animal shelters empty (Frost, 2020; Oakes, 2020; Puente, 2020). Additionally, outdoor recreation was affected in number of trips, location of trips, and monetary value of trips when social and physical distancing policies limited access to other forms of entertainment (Derks et al., 2020; Landry et al., 2020; Randler et al., 2020; Rice et al., 2020; Howarth et al., 2021).

In the United States, equids are typically viewed by the public as straddling the line between livestock and companion animals. The USDA designates equids as livestock though equine owners view their animals in several ways which can include livestock, companion animals, performance partners, and family members (Hemsworth et al., 2014; Dubois et al., 2018; Wallace et al., 2019). The use of equids is often placed into five categories: racing, working animals used for farm and ranch work, production animals used for breeding, competition equids used in various riding styles/disciplines for sport, and/or as recreational or companion animals. Considering this unique position as a source of labor, sport, and recreation, little is known about how the pandemic impacted the equine industry and its participants. To understand how COVID-19 impacted equine owners and leasers this study aims to address three objectives: (1) to understand the financial strain the COVID-19 pandemic had on equine owners and leasers; (2) to investigate what the market for equine care would bear should prices for essential care increase; and (3) to identify the factors contributing to the willingness to pay for equine essential care.

## Materials and Methods

To address study objectives, an online survey (Qualtrics, Seattle, WA) was developed and distributed to equine owners and leasers to gather information about their financial situation as a result of COVID-19 and to estimate their maximum willingness to pay (**WTP**) for essential care for their equid. Specifically, the first objective was completed through summary statistics from questionnaire responses. Using responses from WTP questions, an interval regression estimated the maximum WTP for essential care and the factors that contribute to this reservation price providing insight into equine owner demand for essential care to address the second objective. Finally, to complete the third objective and to investigate what the equine care market would bear in increased costs, a choice experiment was developed.

### Survey population and distribution

After securing University of Tennessee Institutional Board approval (IRB-20-05940-XM), the COVID-19 Equine Financial Stability online survey was sent to equine owners through proprietary email lists, a network of state equine Extension specialists and county agents, and social media platforms (e.g., Facebook, Twitter, and Instagram). All participation was voluntary and limited to one IP address. The data collection period lasted for 4 weeks during the first wave of COVID-19, spanning from July to August 2020. The survey consisted of 26 questions and assessed the impacts of the ongoing COVID-19 pandemic on equine care, ability to pay for and access to essential and nonessential care, and the respondent’s ability to pay for care then and in the future.

Due to the specific population of individuals that own equids, and consequently individuals who either lease, and/or board their equids, respondents were placed into groups with the most limited populations first using skip-logic in Qualtrics. While there may be overlap in survey population classifications, for example, an individual who both owns and leases equids, respondents were presented questions for their designation which was assigned in order as, *Leaser*, *Boarder*, or *Owner*. *Owners* are those who own their equine and are not boarded but kept on their own property. *Boarders* are those that own their equid and house them in a facility they do not own which may cover essential care or not. *Leasers* are individuals that lease an equid regardless of where the animal is housed. For example, the equine owner who also leases an equid was placed in the leasers group as it was expected to have a more limited population to draw from compared to owners. Distinction between *Owners, Boarders, and* Leasers is important as each classification may have differential views of how fluctuating market prices would affect their ability to provide essential care for equids under their care. Individuals were asked to indicate the number of horses they either owned or leased, and were given specified response categories of 0, 1, 2–4, 5–9, 10–19, or 20 or more. All questions on costs were related to all horses owned rather than for individual horses to reduce burden on respondents who may know their full bill rather than itemized per horse.

In an attempt to further classify *Leasers* and *Boarders,* categorization of full, partial, or free lease or board were asked of each respondent with an accompanying definition to reduce individual interpretation. Specifically, free lease was defined as no cost to the leaser for use of the animal, equids may or may not be shared with another person; partial lease which includes some cost associated for use of the equid, animal is shared with another person; and full lease defined as cost permits sole use of the equid by the leaser. For *Boarders,* free board cost was defined as no cost to the boarder for facility use, feed, or daily care, excluding veterinary and farrier cost; partial board was defined as some cost for facility use, feed, or daily care, excluding veterinary and farrier cost; and full board was descriptive all cost for facility use, all feed, and daily care, excluding veterinary and farrier cost. An “Other” response option was provided for both lease and board questions with a free text entry box and responses were binned into existing classification according to an individual’s responses where appropriate.

Essential care was defined as providing feed, water, activities to minimize pain, fear or distress, and daily care such as turnout, while nonessential care includes riding, riding lessons, training, club meetings, and/or boarder access for those not responsible for feeding and daily care.

### WTP and model development

To assess WTP, each respondent was presented with double bounded, dichotomous choice questions based on their responses to two questions; their current monthly cost of care, monthly boarding cost, or leasing fee (Hanemann et al., 1991; Tonsor et al., 2013; Kibler and Pendell, 2018) and a question asking the number of horses owned and kept at home, owned and boarded, and leased. Respondents were first presented with a question based on their group. For example, the *Owner* question was: “Approximately how much do you pay to feed all of your horses (water, hay/forage, and concentrate only) each month on your own property?” The same question was adapted and provided to boarders and leasers for an approximate cost for their lease or boarding situation per equid per month. These values were recorded and used later to reflect a potential change in cost relative to the respondent’s current monthly cost. Owners, boarders, and leasers were asked to evaluate several hypothetical scenarios where feed costs increased and respond as if they were going to spend real money to pay them, which aided in establishing the upper and lower bounds (Table 1). They were asked “Would you be willing to pay $C_1_ per month to feed your horse(s) the same amount of hay and concentrate you currently feed?”, where *C*_1_ was a calculated value reflective of a 10% increase to each respondent’s initial cost of care. Participants were prompted to respond yes or no. Those answering yes were presented with a follow-up cost ($C_{2}^{Y}$), which was an increase of 11% to 20% to their current cost of care randomly selected using the Qualtrics native Mersenne Twister algorithm (Qualtrics, 2015) consistent with the literature (Kibler and Pendell, 2018; Thompson et al., 2018; Campbell et al., 2021). Those answering no to the initial WTP question were presented with a randomly selected value between 1% and 9% higher than their current cost of care ($C_{2}^{N}$). The cost increases were based on anecdotal cost increases and expert equine industry opinions at time of survey design to reflect a range of possible and realistic cost changes. Also, it is important to note that while respondents were asked about the same quantities of feed and hay, a no response indicates a trade off in quality, quantity, or other decision where the respondent is unwilling to pay the price, but their actual trade is beyond the scope of this analysis. Survey WTP questions can be found in the Supplementary Material.

**Table 1.** Willingness to pay response sequence intervals and respective percent of survey responses

Response sequence	Lower bound	Upper bound	% of owners	% of boarders	% of leasers
Yes, yes	$C_{2}^{Y}$ : 11%–20%	.	71	6	65
Yes, no	C_1_: 10%	$C_{2}^{Y}$ : 11%–20%	13	5	10
No, yes	$C_{2}^{N}$ : 1%–9%	C_1_: 10%	3	23	6
No, no	.	$C_{2}^{N}$ : 1%–9%	13	66	20

*Note* Unrestrained values are presented as periods.

A respondent *i*’s true WTP can be modeled as a function of a series of explanatory factors (*X*_*i*_) (Tonsor et al., 2013) using equation. 1:


$$\begin{array}{l} WTP_{i}=X_{i}\beta +\varepsilon_{i}\; \end{array}$$ (1)

where β is a vector of coefficients and $\varepsilon_{i}$ is the independent and identically distributed (iid) normal error term with assumed standard deviation of σ. To calculate the WTP, the responses to the dichotomous choice question are used to create intervals around the costs the respondents were presented, where true WTP for each respondent would lie between their upper and lower bound: *C*_*i*,lower_ ≤ WTP_*i*_ < *C*_*i*,upper_. For example, a double yes response would have an unrestrained upper bound and a lower bound of $C_{2}^{Y}$ and a double no response has $C_{2}^{Y}$ as the upper bound and the lower bound is unrestrained.

Combining the true WTP model with the dichotomous responses presented, an estimate of the WTP can be made with an interval regression (Cameron and Quiggin, 1994; Tonsor et al., 2013; Campbell et al., 2021). The log-likelihood function (**LLF**) (equation 2), assuming a standard normal distribution function (Φ) is:


$$\begin{array}{l} LLF=ln\left[\Phi \left(\frac{C_{i,upper-X_{i}\beta }}{\sigma }\right)-\Phi \left(\frac{C_{i,lower-X_{i}\beta }}{\sigma }\right)\right]. \end{array}$$ (2)

This function allows an estimate of the WTP by considering the factors and owner/boarder/leasers characteristics that contribute to its value. The expected mean WTP is then estimated using these coefficients and the sample averages for the independent, explanatory variables (Tonsor et al., 2013).

For boarders and leasers, the respondent’s current care situation and cost of care is included in the analysis (*CurrentCareCosts*), broken out for each of the three categories. The WTP questions were respondent classification specific: *Leasers* were asked what type of leasing situation they had which were free, partial, full, and “other.” Due to limited observations in modeling, the categories are included as binary variables in the model as *Partial Lease* and *Full Lease.* Similar to *Leasers, Boarders* were asked what characterized their boarding situation: free, partial, full, or “other” and are used as binary variables in the analysis as *Partial Board* and *Full Board*.

To account for the number of equids under the respondent’s care, responses from the survey were placed into a series of binary variables related to a range of the number of animals owned or leased. The binary bins included in the analysis included 1, 2 to 4, and 5 or more equids. Additionally, to account for differences in the value of the animal, training, and use, disciplines are used as explanatory factors. These binary variables include *sport disciplines, ranch/western related disciplines, or other recorded disciplines*.

### Impact of COVID-19 on financial stability

Part of the COVID-19 Equine Financial Stability survey focused on the financial stability of respondents and any disruptions in care resulting from COVID-19. To account for how financial stressors may impact a respondent’s WTP for increases in their care costs, the variables *COVID Impact: Less Stable* and *COVID Impact: More Stable* were assessed to indicate whether a respondent had an impact in their financial stability resulting from COVID-19. Related to this concept, respondents were asked if they were considering selling their equid or stopping their lease due to COVID, where response options included yes (e.g., *Sell Post COVID: Yes*), maybe (e.g., *Sell Post COVID: Maybe*), or no (not listed, in the baseline) to allow for differences in WTP resulting from a forward-thinking perspective. For example, a respondent may be considering selling their equid because of COVID due to financial strain and would not likely be WTP any increase.

Finally, to capture respondent demographics several binary variables were included. *South* distinguishes whether respondents were living in the southern United States as defined by the U.S. Department of Commerce’s Census Regions (2013) including Alabama, Arkansas, Delaware, Florida, Georgia, Kentucky, Louisiana, ­Maryland, Mississippi, North Carolina, Oklahoma, South Carolina, Tennessee, Texas, Virginia, and West Virginia. Respondents residing outside of the South region were included in one category, Other Region, and included ­Census regions West, Midwest, and Northeast (USDC, 2013). Participants reported annual household income across 6 ranges: $0–$25,000, $25,001–$50,000, $50,001–$75,000, $75,001–$100,000, $100,001–$150,000, and greater than $150,000. Respondent annual gross household income (***AHI***) were assigned if their income was above $75,000 (ranges including $75,001–$100,000, $100,001–$150,000, and greater than $150,000). The age of the respondent was also included as age: younger than 35 (including ranges 18–24 and 25–34) and older than 35 (including ranges 35–44, 45–54, 55–64, 65–74, and 75–84). These demographics were included to account for individual WTP heterogeneity. Sex was not included due to limited variability.

### Statistical analysis

Data were summarized and analyzed in Stata 16.1 SE (StataCorp, 2021) to determine mean, minimum, maximum, and standard deviation of all variables. The percent of observations was calculated by dividing the number of observations from the group in question by the total number of observations in the group $\left(\frac{1}{n}*\underset{i}{\sum }\;n_{i}\right)$ (Table 1). Mean testing was performed in Stata to compare price values differences between groups. Mean comparisons used student *t*-tests where significance was determined at *P* ≤ 0.05 and trends identified when *P* ≤ 0.10. Where appropriate, data are expressed as mean ± SE. To estimate WTP, interval regression models were estimated with Stata 16.1 SE (StataCorp, 2021) and significance was determined at *P* ≤ 0.05 and trends identified when *P* ≤ 0.10. Robust standard errors were used to account for heteroskedasticity in the data and ensure consistent and efficient estimations. Results are presented where possible as coefficient ± SE.

## Results and Discussion

The current study expands on the nascent literature on the financial burden of equine care in the face of financial distress in situations like COVID-19 by estimating equine owner/leaser WTP for essential care and the amount they would be willing to pay above their current cost for care/board/lease. Additionally, this study investigates equine owner financial impacts due to COVID-19 and surveyed owners during the first wave of the pandemic which provides a depiction of the financial status of equine owners/boarders/leasers in a state of uncertain information. With ambiguous personal financial situations such as unemployment or job instability and rising costs to equine owners, boarders, and leasers related to feed, cleaning and disinfection, veterinary care, or supplies, understanding what the market may potential bear provides a deeper understanding of the equine market and how financial distress may affect the participants and market trends.

### Participant demographic and reported financial summary

Respondent (*n* = 653) demographics reflected owners (*n* = 292), boarders, (*n* = 282), and leasers (*n* = 79) (Table 2). The majority of respondents were from the Southern region (*n* = 556, 73%) while the remainder of respondents were in other regions (*n* = 206, 27%). Females represented the largest population (*n* = 631, 93%) compared to males (*n* = 131, 7%). Participants indicated the following age ranges, 18 to 24 (*n* = 91, 13.3%), 25 to 34 (*n* = 133, 19.5%), 35 to 44 (*n* = 139, 20.4%), 45 to 54 (*n* = 135, 19.8%), 55 to 64 (*n* = 129, 18.9%), 65 to 74 (*n* = 52, 7.6%), and 75 to 84 (*n* = 3, 0.4%). Annual household income was distributed across six ranges including less than or equal to $25,000 (*n* = 49, 7%), between $25,001 and $50,000 (*n* = 135, 20%), between $50,001 and $75,000 (*n* = 112, 17%), between $75,001 and $100,000 (*n* = 122, 18%), between $100,001 and $150,000 (*n* = 134, 20%), and greater than $150,000 (*n* = 113, 17%). Demographics were consistent with recent, previously published literature describing the equine industry within the United States (American Horse Publications, 2018, Heuscheule et al., 2018; Stowe, 2018; Wallace et al., 2019; Jaqueth et al., 2018, Mastellar et al., 2018, Melvin et al., 2020). It is important to note that since many of the respondents in the current study were from the South, a larger, more equal pool of U.S. residents may cause regional differences in prices and preferences.

**Table 2.** Summary of select willingness to pay survey responses

Variable	Description	Obs.	Mean	SD	Min	Max
*Equine ownership*						
Current care costs: Owners	U.S. dollars	292	$406.37	606.40	0	6,000
Current care costs: Leasers	U.S. dollars	79	$190.22	211.19	0	750
Current care costs: Boarders	U.S. dollars	282	$391.42	285.45	0	1,500
Own 1 equid	1 if True; 0 Otherwise	762	0.23	0.42	0	1
Own 2–4 equids	1 if True; 0 Otherwise	762	0.41	0.49	0	1
Own 5+ equids	1 if True; 0 Otherwise	762	0.26	0.44	0	1
Lease 1 equid	1 if True; 0 Otherwise	762	0.09	0.28	0	1
Lease 2-4 equids	1 if True; 0 Otherwise	762	0.02	0.14	0	1
Lease 5+ equids	1 if True; 0 Otherwise	762	0.01	0.11	0	1
Partial lease	1 if Partial lease; 0 Otherwise	762	0.03	0.18	0	1
Full lease	1 if Full lease; 0 Otherwise	762	0.03	0.17	0	1
Partial board	1 if Partial board; 0 Otherwise	762	0.10	0.30	0	1
Full board	1 if Full board; 0 Otherwise	762	0.24	0.42	0	1
Sport disciplines	1 if Equid used for Discipline; 0 Otherwise	762	0.28	0.45	0	1
Ranch/Western related disciplines	1 if Equid used for Discipline; 0 Otherwise	762	0.10	0.30	0	1
Other disciplines	1 if Equid used for Discipline; 0 Otherwise	762	0.14	0.35	0	1
*Financial impact of COVID*						
COVID impact: Less stable	1 if True; 0 Otherwise	762	0.03	0.17	0	1
COVID impact: More stable	1 if True; 0 Otherwise	762	0.56	0.50	0	1
Sell post COVID: Yes	1 if True; 0 Otherwise	762	0.07	0.26	0	1
Sell post COVID: Maybe	1 if True; 0 Otherwise	762	0.09	0.28	0	1
Stop lease post COVID: Yes	1 if True; 0 Otherwise	762	0.01	0.07	0	1
Stop lease post COVID: maybe	1 if True; 0 Otherwise	762	0.01	0.11	0	1
*Demographic characteristics of respondents*						
Female	1 if True; 0 Otherwise	679	0.93	0.24	0	1
South^1^	1 if True; 0 Otherwise	762	0.73	0.44	0	1
Annual household income > $75,000	1 if True; 0 Otherwise	662	0.55	0.49	0	1
Age: Younger than 35	1 if True; 0 Otherwise	679	0.32	0.47	0	1

^1^Respondents residing in the South as defined by the U.S. Department of Commerce’s Census Regions (2013).

The care costs differ by type of respondent and type of situation (e.g., full vs. partial lease). Respondents provided care costs for all horses owned or leased as they were not asked to provide the exact number of horses but rather a range. In addition, given that full, partial, and free lease and board agreements may encompass differential levels of essential care and responsibility, definitions were provided to survey participants to aid in reducing individual response bias. The terms “full,” “partial,” and “free” are commonly used within the equine industry; however, a common misconception exists where free leases are often considered to have no associated cost for care, when in reality the leaser is typically responsible for the entirety of the leased equid’s care (University of Maryland, 2017). Leasing and boarding costs were variable among respondents, where partial lease was the highest monthly cost among lease types ($303 ± $235, min = $0, max = $750, *n* = 26), and full board was the highest board classification ($504 ± $275, min = 0, max = $1,500, *n* = 179) (Table 3). For *Owners* (*n = 292*), the $406 ± $606.40 monthly costs represent the expenditures for feed and hay for all their equids each month (Table 2). While these are not broken down per horse, the mean cost of care can be calculated by number of horses. Owner care costs were $112 ± $118 for one horse, $244 ± $229 for 2 to 4 horses, and $719 ± $891 for 5 or more horses. The standard errors indicate a wide range in prices paid across the survey responses.

**Table 3.** Survey summary of monthly per equid leasing and boarding costs

Leasers					
	Obs.	Mean	Std Dev.	Min	Max
Free lease	28	$50	127	$0	$500
Partial lease	26	$303	235	$0	$750
Full lease	20	$262	169	$0	$750
Other lease	5	$105	150	$0	$325
*Boarders*					
	Obs.	Mean	Std Dev.	Min	Max
Free board	14	$0	0	$0	$0
Partial board	75	$236	151	$0	$650
Full board	179	$504	275	$0	$1,500
Other board	13	$187	262	$0	$750

Across all full, partial, and free *Leasers* (*n* = 79), the monthly lease cost was reported as $190.22 ± $211.19 per equid (Table 1). For *Boarders* (*n* = 282), the mean monthly boarding cost per animal was $391.42 ± $285.45 across all boarding types. Boarding and leasing costs are difficult to quantify, given the variable nature with which board and lease agreements are defined, included services, and frequent lack of formal, recorded documentation of agreements between parties. Boarding costs detected within this study are higher than previously published literature; however, many of these works were completed over a decade ago (Porr et al. 2008). In a study of Virginia boarding operations, pasture only board was reported at $228 per month, which most closely aligns with partial board definition within this study ($302 per month) (Porr et al., 2008). Equine lease agreements parameters and associated costs are even more elusive, whereas land, livestock and machinery leases for agricultural production purposes are more extensively studied (Van Tassell et al., 1997; McCarthy et al., 2007). Considering the recreational aspect of the equine industry, comparative studies assessing leasing opportunities for bicycles, cars, and other depreciable equipment are prevalent (Warmington-Lundström et al, 2020; Zhang et al., 2020). Further studies to classify boarding and leasing parameters, interrelationships between these classifications, and associated costs are warranted, and would aid in determining fair prices for informed business owners and equine care-seekers alike.

### WTP results

The estimated mean WTP for each model (Table 4) is calculated at the mean values for model explanatory variables. Overall, owners were willing-to-pay an additional 25.6 % (*P* < 0.01), boarders 14.2% (*P* < 0.01), and leasers 15.2% (*P* < 0.01) for essential care on average compared to their current care costs. In the face of financial uncertainty driven by large scale events, COVID-19 in this case, prices for inputs and feed can increase due to limited labor, increased transportation costs, and additional costs of doing business (Schmitz et al., 2020; Weersink et al., 2021). Horse owners and leasers may be concerned about rising care costs (American Horse Publications, 2021) however, the results from this study show that the market is willing to bear the burden of some of these costs to maintain quality and continuity of care (WTP increased cost between 14.2% and 25.6%). These values vary over several factors that contribute to individual WTP heterogeneity and are discussed in the following sections. Interestingly, boarders are willing-to-pay less additional relative care costs (14.2%) than owners (25.6%) and leasers (15.2%). Unlike owners, boarders in partial and full board situations are paying for more than the cost of feed in their price for boarding their equid as they pay for use of the facility and its amenities, daily care, and labor, as well as inputs and utilities. Average cost of board for boarders ($391) was lower than owners ($406) but higher than leasers ($190.20) which means that a 10% increase for the average boarder is relatively more than it would be for the average leaser. Additionally, boarders may feel they have access to acceptable substitute boarding facilities should the cost to board at their current location increase.

**Table 4.** Interval regression results for owner, boarder, and leaser models

	Owner model		Boarder model		Leaser model	
	Coefficient	SE	Coefficient	SE	Coefficient	SE
Current care costs	0.00	0.00	−0.00**	0.00	0.00	0.00
Own/lease 2 to 4	−0.02	−0.05	−0.06***	−0.02	0.08	−0.07
Own/lease 5+	−0.07*	−0.06	0.02	−0.05	0.70***	−0.15
Leaser who owns					−0.04	−0.06
Partial board or lease			0.09	−0.1	0.00	−0.07
Full board or lease			0.07	−0.1	0.1	−0.08
Sport disciplines	0.01	−0.04	0.02	−0.02		
Ranch/western related disciplines	0.04	−0.03	−0.01	−0.04		
Other disciplines	0.02	−0.03	0.11***	−0.04		
COVID impact: Less stable	−0.04	−0.11	0.05	−0.05	0.62***	−0.14
COVID impact: More stable	0.07**	−0.03	0.01	−0.02	0.00	−0.05
Sell/stop lease post COVID: Yes	−0.10**	−0.05	0.02	−0.06	0.06	−0.10
Sell/stop lease post COVID: Maybe	−0.04	−0.03	−0.03	−0.03	0.11	−0.07
South	−0.11**	−0.05	0.02	−0.02	−0.03	−0.06
Annual household income > $75,000	0.09*	−0.06	0.04**	−0.02	0.04	−0.05
Age: Younger than 35	0.03	−0.03	−0.03	−0.02	−0.01	−0.06
Constant	0.34***	−0.07	0.08	−0.10	0.07	−0.10
Ln(Sigma)	−1.92***	−0.15	−2.22***	−0.11	−2.27***	−0.23
Willingness to pay for equine care^1^	0.256***	−0.02	0.142***	−0.01	0.152***	−0.02
Wald Χ^2^	27.45**		32.82***		32.63***	
Observations	255		204		44	

^1^Willingness to pay are linear predictions at the means for each respective model.

*P < 0.10; **P < 0.05; ***P < 0.01, Robust standard errors presented.

### Owner interval regression model results

The focus of the COVID-19 Equine Financial Stability Survey relates to how respondents were financially affected during COVID-19. Owners who were more financially stable during COVID-19 were WTP 7% more (*P* < 0.05) than those who were less stable or did not have a financial stability impact. That is to say, that those whose income increased or became steadier during COVID-19 were willing to devote more of their current or increased income in covering essential care costs. Equids straddle the line between production livestock and companion animals and this WTP shows the importance of these animals to respondents as reflected in potential budget reallocations. Respondents who were going to sell their equids as a result of COVID were WTP 10% less (*P* < 0.05) than those who were considering or were not intending on selling, potentially speaking to the decision-making process of selling an equid, where the cost of upkeep may have already been financially taxing. During unprecedented events such as COVID-19, animal care can bear the burden of owner financial stress. These results show a resilience and dedication of equine owners in continuing care even at potential personal reductions. Further, work completed by Huseman and others indicated that reduction in in-person horse shows during the early pandemic timeframe may have reduced overall spending for extracurricular equine activities. With a reported $991 spent per in-person horse show, and a reduction of 4 shows within early pandemic restrictions, it is possible that disposable income for many equine owners was reallocated to essential care (Huseman et al., 2021).

Several additional factors significantly contribute to an owner’s WTP. Owners with more than five equids owned and not boarded are WTP 7% less (*P* < 0.1) than those owning less than five equids. The majority of owners care for two to four equids, which aligns with previously reported industry ownership per person (Melvin et al., 2019; Wallace et al., 2019). The survey used relative changes in costs, but the absolute change in cost for an owner with five or more equids would be substantially more than if fewer animals were owned. In considering larger operations, it could be ­possible that owners were not WTP as much for additional care if they produced their own hay or maintained their own pastures and were not considering their own labor expenses. One concern during financial hardships is an increase of equids being sold or abandoned. Results indicate that the horse market should not expect a substantial increase in the number of equids listed for sale as a result of the financial hardships from the pandemic and that some increase in the cost of care will be tolerated by this population. These findings are consistent with the 2021 Equine Industry Survey where 90.3% of respondents indicated expectations of owning/managing the same or more horses in the future (Stowe, 2021).

In terms of demographics location and income had significant effects on owner WTP. Respondents in the southern United States were WTP 11% less (*P* < 0.05) than the respondents in other regions. This could reflect differences in access to year-round forage, longer forage periods, or climatic differences in the south to other regions. It may also reflect the regional value of money, where due to relative cost of living differences, the additional care costs would be perceived differently. Those with AHI greater than $75,000 were WTP 9% more (*P *< 0.1) than those making less, speaking to the potential differences in disposable income. While we did not question the value of equids owned, it is possible that this AHI level owns more valuable equids which could account for their WTP more for their essential care during a finically distressing situation like COVID-19.

Researchers in the United Kingdom surveyed equine owners in March of 2020 to understand the impact of the pandemic on their ability to interact and manage their equids (Williams et al., 2020). Their findings showed differences by facility type where owners with equids kept in partial and full boarding situations experienced major impacts to the daily management of their equids, consistent with results by Hockenhull et al. (2021) and Merkies et al., (2020). Equine owners who kept their animals at home and those who pasture board felt the least disruption to daily management of their equids. These two groups would have access to their animals regardless of COVID-19 physical and social distancing polices in place. Across all facility types, approximately a third of survey respondents showed concern for their future ability to provide for their animal’s essential needs including medical, farrier, feed/forage costs, and boarding costs.

With uncertain personal financial situations such as unemployment or job instability and rising costs to horse owners and leasers related to feed, cleaning and disinfection, veterinary care, or supplies, understanding what the market may potential bear provides a deeper understanding of the equine market and how financial distress may affect the participants and market. Further, it is possible that the timing of the study (July–August 2020) was reflective of early-phase COVID response where individuals felt more secure in their financial situation and may have assumed the pandemic would cease in the near future. A current assessment outside of early phase COVID response may be useful to determine if current perspectives echo initial responses. Unfortunately, contact information was not captured from survey participants to allow for data collection from the same population.

Determining the difference between an individual’s ability to perform and pay for essential vs. nonessential care is an important distinction, especially for the animal’s welfare and exercise. During early phase COVID restrictions, many facilities permitted access to animals for essential care ­reasons, but often nonessential care was restricted (Greene et al., 2020). A survey administered by Hockenhull et al. (2021) in the first few days of the emerging pandemic examined biosecurity practices and nonessential care at equine facilities in the United Kingdom. Findings showed that while some respondents stopped riding altogether, others took steps to minimize their risk of COVID-19 exposure through personal disease mitigation measures and limited unnecessary health risks while riding such as avoiding dangerous riding locations (e.g., loud or busy roads) or dangerous riding situation (e.g., jumping or riding young or nervous animals). Similar findings on nonessential care were found in a study conducted in Ontario, Canada (Merkies et al., 2020) which administered a questionnaire in July of 2020 to lesson barns and training facilities in the area. The authors report approximately 45% of those surveyed reduced the amount of access to their facility and nearly 50% of respondents reduced the size of riding lesson groups and disinfected tack to reduce the risk of spread of COVID-19 at their facility. Further, Hockenhull and Furtado (2021b) concluded that equine owners in COVID lockdown were more observant of management impositions placed on equids in confinement and may have addressed shortcomings in essential and nonessential care within their operation. While these considerations were not formally addressed within the current study, it is important to recognize that alterations in nonessential care access may have impacted reallocation of financial resources to pay for essential care seen within the current study outcomes.

### Boarder interval regression model results

Boarders are in a unique position that they own their equids but board them at a facility, which could affect the equid-to-owner relationship and increases the monthly financial liability of ownership. On average the current care costs significantly impact boarder’s overall WTP for increase in care cost, where a dollar increase in current care cost decreases the WTP by 0.01% (*P* < 0.05). For instance, boarders with $100 difference in current care costs, those paying more would be WTP 1% less than those paying less for increased care costs.

Boarders with two to four equids have a significantly lower WTP (−6%; *P* < 0.01) than those who only boarded 1 equid, with the majority of respondents caring for two to four equids. These boarders may be WTP less due to the increase in total bill associated across all their animals. Their WTP did not differ by type of boarding situation, this could be that the terms of boarding vary across boarders and facilities, with many add on services that may mitigate any effects of boarding situation on WTP. There were differences in discipline in that those boarders whose equids were engaged in *Other Disciplines* were WTP 11% more (*P* < 0.01) than those engaged in no or other specified disciplines. This may indicate some financial incentive in the discipline earnings.

COVID-19 effects were not significant for boarders. This was surprising as boarders directly pay for care and maintenance for their animals. However, this may be indicative of the stability or socioeconomic situation that many boarders are in, such that COVID-19 may not have had a substantial or significant impact on their day-to-day. Further, these results may also be reflective of a disconnect between boarders and knowledge of equine essential care costs, facility upkeep and costs contained within monthly boarding charges. Future work could evaluate the implication of COVID-19 on facility owners, that is, equine care supply side, to understand their responsiveness to short and long-term price increases and how they transmit through the equid care market.

### Leaser interval regression model results

Contrary to owners, leasers with more than five equids were WTP more for essential care (70%; *P* < 0.01), which may reflect larger leasing operations demand for equids. These may be trail riding businesses or riding classes that need the animals for business continuity and are WTP more in the face of increasing care costs than the more personal demand of leasers with fewer equids. It should be noted that in general 76% of leasers were leasing one equid. In addition, there were not significant differences between leasers who solely leased (46% of the leaser sample) and leasers who also owned in their WTP.

Financial stability in the face of challenges imposed by COVID-19 had a seemingly inverse relationship for leasers. Those who reported themselves less financially stable as a result of COVID-19 were WTP 62% more (*P* < 0.01) than those who reported being more financially stable or had the same financial stability as pre-COVID. The relative difference between how respondents were impacted financially and their WTP may relate to why some would choose to lease a horse compared to buy a horse, when they may be starting out or cannot afford to own an equid. If an individual is less stable, lack of disposable income is not implied, but could relate to consumer surplus in leasing markets. First order interaction effects were tested and were not significant.

The majority of *Owners* (71%) and *Leasers* (65%) responded yes to both WTP questions, whereas the majority of *Boarders* (66%) responded no to both questions. These differences will be explored more in the discussion section, but this could indicate more financial pressure on *Boarders* over *Owners* or *Leasers*.

## Conclusion

Financial distress can cause disruptions in care for companion animals and livestock. COVID-19’s global personal and business disruptions were varied and substantial. Supply chain disruptions impacted economies and unemployment as well as agricultural production such as feed and hay. Transportation shortages limited movement of available stocks which increased the prices for goods and services. There were reductions in recreation activities and access to recreational activities in many areas due to restrictive policies implemented to reduce spread of COVID-19. These varied disruptions affected equine care and activities considering equids exist along the spectrum of livestock and companion animals which places them in the center of a financial quandary where owners and leasers care of their animal is paramount even in the face of financial stress.

The value of an early pandemic survey is that it provides an understanding of how equine owners, boarders, and leasers were responding to the emerging disease amidst an uncertain future. Overall, this study found that equine owners, boarders, and leasers were not substantially financially impacted by COVID early in its emergence and that each group was willing to pay at least 14% or more for their current care cost situation. This implies that the market is able to bear the potential changes in costs. The inelastic nature of equine care implies a trade off in budget allocation when faced with higher care costs. Future work is warranted to evaluate current status of equine owners, boarders, and leasers to determine how late pandemic effects compare to early phases responses.

## Supplementary Material

skad091_suppl_Supplementary_Material

## Abbreviations

AHI: annual gross household income
CDC: Centers for Disease Control
COVID-19: SARS-CoV-2
US: United States
WHO: World Health Organization
WTP: willingness to pay

**Mathematical Abbreviations**
*C*_1,2_: costs shown in survey for first or second WTP question
LLF: log-likelihood function
Φ: standard normal cumulative distribution function
*X*_i_: matrix of explanatory variables
β: vector of coefficients
