# Supplementary material for: Financial strain of COVID-19 and its impact on willingness-to-pay for equine care
Source: J Anim Sci. 2023 Mar 26;101:skad091. doi: 10.1093/jas/skad091 (PMC10112674; doi:10.1093/jas/skad091)
Supplement: skad091_suppl_Supplementary_Material [file skad091_suppl_supplementary_material.docx]

**Appendix**

Survey Design for eliciting Willingness to Pay

**Owners:**

We would like you to consider the monthly cost to feed your horse(s) in a hypothetical situation. Assume this hypothetical situation does not have any impact on any other care costs associated with your horse(s) outside feeding cost.
 
Please respond to each scenario as true-to-life as possible. Select the choice you would make should real money be on the line as your responses may direct future outreach and extension programs.

Would you be willing to pay $X*1.1 per month to feed your horse(s) the same amount of hay and concentrate you currently feed?

Would you be willing to pay $X*(1+Random%Higher) for the same amount of hay and concentrate you currently feed?

Would you be willing to pay $X*(1+Random%Lower) for the same amount of hay and concentrate you currently feed?

**Boarders:**

We would like you to consider the monthly rate you charge to board a horse at your facility in a hypothetical situation. Assume this hypothetical situation does not have any impact on the type of care you provide boarded horses.
 
Please respond to each scenario as true-to-life as possible. Select the choice you would make should real money be on the line as your responses may direct future outreach and extension programs.

Should cost to operate your facility change, including feed costs, do you think current boarders would be willing to pay $X*1.1 for the same boarding situation?

Would current boarders be willing to pay $X*(1+Random%Higher) per month for the same boarding situation?

Would current boarders be willing to pay $X*(1+Random%Lower) per month for the same boarding situation?

**Leasers:**

We would like you to consider the monthly cost to lease your horse(s) in a hypothetical situation. Assume this hypothetical situation does not have any impact on any other care costs associated with your horse(s) outside your lease rate.
 
Please respond to each scenario as true-to-life as possible. Select the choice you would make should real money be on the line as your responses may direct future outreach and extension programs.

Would you be willing to pay $X*1.1 per month for the same leasing situation you are in right now?

Would you be willing to pay $X*(1+Random%Higher) per month for the same leasing situation you are in right now?

Would you be willing to pay $X*(1+Random%Lower) per month for the same leasing situation you are in right now?
